# Retroversion of the Unimpregnated Womb

**Published:** 1872-10

**Authors:** A. Reeves Jackson

**Affiliations:** Surgeon-in-Chief of the Woman’s Hospital of the State of Illinois; 785 Michigan Ave.; Chicago


### Chicago Medical Journal.

A MONTHLY RECORD OF

*Medicine, Surgery and the Collateral Sciences.*

EDITED BY J. ADAMS ALLEN, M.D., LL.D.; AND WALTER HAY, M.D.

VOL. XXIX.—OCTOBER, 1872.—NO. 10.

### Original Communications.

ARTICLE I.—*Retroversion of the Unimpregnated Womb.* By A. REEVES JACKSON, M.D., Surgeon-in-Chief of the Woman's Hospital of the State of Illinois. (Read before the Chicago Society of Physicians and Surgeons, Sept. 2, 1872.)

The practice of medicine would seem to require, more than any other art, a harmony of opinion among its professors, for there is no other in which error is of so much consequence, or where doubt and uncertainty entail so much anxiety. Yet the history of medicine is full of schisms and discrepancies, and the sages of our art have left us a heritage of opposing sentiments upon almost every question of theory and practice. To no one subject do these remarks apply more fitly than to that of retroversion of the womb.

Although allusions were made to this displacement by many of the older medical writers, it was first accurately described by Hunter, in 1754. Since that time there is scarcely a writer on midwifery who does not mention it; yet notwithstanding this frequent notice of the disorder, and the great amount of research that has been devoted to it, there is hardly a disease in relation to which there exists a greater diversity of opinion as regards its importance, its pathology, and its management. While one set of practitioners view it as an accident of the most serious kind, and as giving rise to all the distressing symptoms that are known to accompany it, another, equally competent, regard it as a matter of little concern or importance. We are told, on the one hand, that the reposition of the displaced organ is a primary and essential point in the treatment, without which a cure is impossible, and, on the other, that if we direct our efforts to the removal of the accompanying engorgement and inflammation, we will have met all the therapeutical requirements, and that these conditions being removed, the organ will restore itself, or at least give no trouble. Now these are diametrically opposing views, and, as in most cases where extreme opinions are held upon any subject, the truth will be found to lie somewhere between.

We all understand how these things occur, and how it is that doctors so proverbially disagree. Whether consciously or unconsciously, every observer of natural phenomena forms a theory; consciously or unconsciously his subsequent facts are too often made to bend to that theory; those that are not sufficiently pliable for this purpose, are very likely to be rejected. He finally takes a position and forms an opinion, perhaps announces it; pride urges him to sustain it, and the result is that, unknowingly perhaps, he is dishonest—dishonest with himself and in his relation to science. No one objects to the truth merely as truth, but it must come in and take its place quietly, and without making disturbance, if it would be welcomed. But if it come as a waster, changing, displacing, and moving us from positions in which we have found ourselves comfortable, we are apt to regard it as an intruder, and either do not admit it at all, or do so with reluctance. In medicine, all experience shows that it is very difficult to avoid empiricism, and not become dogmatic.

In making these remarks I do not wish to be understood as condemning the mental processes which so often lead to these confusing results. Indeed, I regard intelligent thought itself upon any branch of human knowledge, as impossible, without the formation of hypothesis or theory. We naturally desire to understand that which we observe. The facts we learn by mere perception are only the stimuli to thought. These facts are compared with those that we already know, and from this comparison results an inference or conclusion, and this conclusion is a theory.* The error, therefore, does not consist in forming the theory, but in the assumption that the observations upon which it is based are complete and embrace all the facts, and that its correctness is not to be tested by subsequent experience.

Before entering upon the subject of retroversion of the womb, let us recall for a moment the natural position of the organ, and its relation to the surrounding parts. Weighing from one to two ounces, it occupies nearly a central position in the pelvis, in contact only with soft yielding tissues, in the midst of which it is loosely suspended. Its fundus is about midway between the symphisis pubis and the promontory of the sacrum, and a little below a line drawn between those points. Here it is sustained by all the pelvic contents, and in speaking of the uterine supports, I think we ought to regard the pelvis as a whole, for although different organs and tissues each have their own part of the work to perform, they each contribute to a general result. Thus, not only is the uterus held in position by the round and broad ligaments, the anterior vaginal column, the structures of the perineum, etc., but also by its relations to and connections with the bladder, the rectum, and all the fasciæ, ligaments, and other parts and contents of the pelvic cavity. But all these do not by any means *fix* the womb in its position. The fundus, particularly, has considerable freedom of motion, especially forwards and backwards. The long axis of the organ and the direction of its cavity are identical with that of the brim of the pelvis, and are represented by a line drawn from the umbilicus to the os coccyx.

Unquestionably the strongest guard against retroversion is found in the round ligaments. And yet from the oblique direction of their attachment, they readily permit the womb to assume a vertical position—that is, one in which the axis of the organ corresponds with that of the whole body. But it cannot go beyond that position without subjecting these ligaments to a certain amount of traction. Hence, I think we may regard any case in which the fundus is carried backwards beyond such vertical line, as one of retroversion. But the round ligaments are not the only organs that prevent retroversion. Indeed, nature has been lavish in fur-

* Laycock on Medical Observation, page 28.

nishing means against the occurrence of this accident. There are, besides the cervico-sacral ligaments below and behind, the soft elastic intestinal cushion in Douglas' cul-de-sac, and the peculiar attachment of the broad ligaments, all fortifying the uterus in this direction.

It was at one time thought that the unimpregnated womb was not liable to retroversion, and all the earlier notices of the displacement had reference to the pregnant organ. Now, however, since gynæcology has come to be regarded as a legitimate field of practice—a field in which honest reputable men may labor without risk of excommunication—it is well known not only that it may occur independently of pregnancy, but that it does so very frequently. Indeed, my own experience leads me to believe that it is one of the commonest derangements of the female generative organs. In view of this frequency of occurrence, the great distress which usually accompanies it, its effect in preventing conception and in producing abortion, we must admit its importance and its claims upon our attention.

Much of the variance of opinion that we find among authors in regard to the relative frequency and gravity of this affection, may be reconciled, I believe, by a more accurate knowledge of its pathology and its complications, and it is of the highest importance to us as practitioners, that our ideas should be entirely clear, or at least as clear as the nature of the subject will admit. All correct treatment must be founded on correct views of causation and pathology, and if these latter be erroneous, so likewise will be the former.

Retroversion of the womb is defined to be that deviation of the organ in which the fundus is carried backwards and downwards from its normal position, and the cervix forwards and upwards. When the displacement is sufficiently marked, the organ will be found occupying a position between the vagina and rectum, and its neck or mouth will be elevated in an opposite direction, and will be found in the anterior and superior part of the pelvis, and behind or even above the symphisis pubis. This change in the situation of the uterus may be induced suddenly, or a considerable time may elapse before it becomes established. I have seen two cases in which it was almost instantly produced: in one, by a severe blow across the back while stooping; in the second, by attempting to lift a heavy weight. In both instances the symptoms were violent and alarming. There was stoppage of the urine and feces; pains resembling those of labor, but more continuous, and a disposition to syncope; and in both cases these symptoms rapidly subsided on the replacement of the organ.

When the displacement takes place more slowly, the symptoms are much milder, and only acquire intensity when the deviation has reached an extreme degree. During the progress of the change, and sometimes even after the change has taken place, the sufferings of the patient are not extreme. We find her complaining, perhaps, of a difficulty in urinating, with an increased desire to do so; a painful dragging about the hips, loins and thighs, and occasionally a forcing, bearing-down pain. In most cases there is also present more or less leucorrhœal discharge.

The first point to which I desire specially to ask your attention relates to the cause or causes of the symptoms which have been enumerated as accompanying retroversion. Are they produced by the displacement? Or, are they caused rather by other accompanying pathological conditions? Those who believe that the displacement itself is the cause of the pains and leucorrhœal discharges observed in these cases, explain these results in two ways: 1, from the dragging upon the ligaments of the uterus—that is to say, upon the parts which sustain the organ in position; and 2, from the pressure exerted by the displaced viscus upon the various organs contained in the pelvis. To my mind this explanation seems most inadequate and unsatisfactory. When we consider the natural laxity of these uterine supports, and the great freedom of motion they permit to the fundus; and when we further consider the limited sphere within which the motions of the organ are confined even in extreme cases of retroversion, there seems to be little reason for believing in the alleged painful traction upon the ligaments by the displaced womb. Again, these displacements are frequently caused by the development of tumors in the uterine walls, and in these cases, although the retroversion may exist in a marked degree, the patients do not commonly seek for advice unless impelled to do so by reason of hemorrhage or the inconvenient increase in the size of the abdomen.

As regards the pressure exerted by the displaced womb upon the neighboring organs, it is readily seen that this cannot be the cause of the symptoms attributed to it, for two reasons: first, because the volume of the uterus, simply displaced is not increased, and is not sufficient to cause actual compression of the pelvic organs; and secondly, because this pressure is quite easily borne in a great number of cases where tumors much larger than the displaced uterus occupy the greater part of the pelvic cavity, and inevitably compress the contained viscera.

I have now under my charge a lady who has a fibrous tumor of the womb, sufficiently large to fill the entire pelvic cavity, and to reach almost to the umbilicus. It is only with the greatest difficulty that I can introduce a finger on either aspect of the tumor between it and the pelvic walls, and the flattened, ribbon-like form of the voided feces plainly indicates the pressure to which the rectum is subjected, and yet this patient only seeks medical assistance in consequence of occasional hemorrhage. For these reasons and some others which will appear presently, I am inclined to regard retroversion of the unimpregnated womb, simple and unconnected with any lesion of tissue, as ordinarily a harmless affection, provided it occur with sufficient slowness to accustom the involved parts to the change. In cases where it occurs suddenly, as in those cited, the parts are surprised, so to speak, and they resent the disturbance.

The great difference observed in the character and severity of the symptoms in acute and chronic cases of retroversion, would be very surprising if it were not that clinical experience furnishes us with an abundance of instances proving the tolerance to injurious impressions which may be induced in the human body, provided it be very gradually subjected to their influence. We have a familiar example of this in the enormous quantities of opium which may be safely taken by one long addicted to the habit; also, in the facility with which a person having chronic ophthalmia may expose his eyes to a degree of light which would be unendurable to one whose eye had been inflamed only a few hours. In expressing this opinion of the harmlessness of retroversion, I am quite aware that it differs from the one commonly held, and the reasons which seem so conclusive to my mind may not seem at all so to others. Nevertheless I bespeak for them your consideration.

In addition to the inadequacy of simple retroversion to produce important symptoms, already alluded to, we have the fact that replacement of the organ does not usually give relief, and in many cases all mechanical attempts at reposition and retention, actually increase the patient's distress.

In the earlier years of my practice, acting under the belief that in a case of retroversion of the womb my clear duty was to rectify the displacement, I proceeded at once to restore the organ to its normal position, and then used such mechanical retentive means as seemed applicable. Hodges' lever pessaries and Meigs' ring were my favorites for this purpose. All this was done without reference to any existing complications, or to the duration of the malposition. My degree of success was not gratifying. Occasionally a patient would report herself as more comfortable, but in a very much larger number the local distress was so increased that I found myself obliged to remove the favorite and substitute some other form of support, which in its turn was removed to be followed by a third, and so on. It was a long time before I succeeded in unlearning what I had been taught upon this subject, but when I did so I ceased using (at least under the same circumstances) either the favorites or their substitutes, with great advantage to my patients.

Again, in a very large number of the cases of retroversion we are called upon to treat, there is present some structural disease of the womb, which is quite sufficient to produce all the symptoms present, independently of the displacement. And without undertaking at present to determine the relation which exists between the malposition and these organic changes, I will call your attention to the more frequent and important of these latter.

And, first, a very frequent accompaniment of retroversion is engorgement of the uterus. The organ is found to be enlarged, heavy, congested, and painful to the touch. In some instances this enlargement affects only a portion of the organ, as the body or the cervix, but usually the entire organ is involved, the walls being thickened, the cavity enlarged, and its orifice open. Sometimes the lips are swollen, often lobulated, projecting into the vagina, red and granular in appearance, and bounded by a distinct line marking the division between the mucous membrane of the vagina and that of the uterus—the condition, in short, which has been so frequently described as ulceration. In these cases we have precisely the symptoms that are present in cases of inflammatory engorgement without displacement, viz.: pelvic and hypogastric pains, leucorrhœa, and sympathetic disturbance of the functions of the neighboring organs. Although this condition of engorgement is a very frequent complication of retroversion, it is not by any means a constant one, and I have known at least two cases of well marked retroversion in which the uterus was decidedly undersized.

Associated sometimes with this hypertrophied condition of the womb, but existing also without it, is a chronic form of inflammation of the lining membrane of the cavity—either cervical or corporeal endometritis, or both—and then we have to add to the list of symptoms the various nervous disorders, menstrual irregularities, pains and leucorrhœa, which characterize these conditions.

Finally, a very frequent complication of retroversion is retroflexion of the womb. So commonly indeed are they found co-existent, that it is quite customary among systematic writers to classify and treat them together. But there is a wide difference between these conditions pathologically, and there should be clinically. Retroversion, as before stated, implies mere deviation of position, while retroflexion involves structural change, and hence its presence, like the other conditions mentioned, adds a new element of disorder, and a new cause of discomfort. The abnormal state now alluded to should not be confounded with incurvation of the organ, which I regard as a quite common and quite harmless condition. When it is present the entire organ is curved, and the bending of the canal corresponds exactly with that of the walls. But in true flexion of the womb there is a doubling or folding of it upon itself at some one point, usually at or near the junction of the cervix with the body. Bearing in mind that the uterus is an organ with thick walls and a narrow cavity, we can understand how it is that in a case of flexion the organ may present a merely curved outline, especially on the side *from* which the flexion proceeds, while the effect on the canal is to cause an actual compression and obstruction, and most of the cases of sterility and dysmenorrhœa which are associated with retroversion are those in which there is flexion also.

Having now considered these complications or accompaniments of retroversion, it seems proper to inquire into the relations which exist between them and the displacement, and to ascertain, if possible, which is first in order of occurrence. This inquiry is full of interest, and its solution must have an important bearing upon our therapeutics.

Among the means which the Creator has provided to guard against retroversion, the broad ligaments were enumerated. The manner in which these organs effect this object is peculiar. Their attachment to the womb is a very extensive one, reaching from the superior angle of the organ on each side as far down as the insertion of the vagina, and this broad connection gives rise to important results. When retroversion occurs, these ligaments must necessarily be twisted upon themselves, the degree of torsion depending upon the degree of displacement. When this latter is slight, the effect upon the ligaments will be slight also, but in those extreme cases in which the fundus of the womb must describe in its passage an arc of a circle equal to 160 degrees, and is found low down in the hollow of the sacrum, the broad ligaments must undergo a proportionate degree of torsion. This obstructs the venous channels, and this obstruction naturally gives rise to ovarian and uterine congestion, hypertrophy, hyperæsthesia, and menstrual disturbance.* But notwithstanding the presence of these conditions in retroversion might be explained on the assumption of the displacement being the primary disorder, I do not believe that ordinarily it is so. I regard this latter as secondary in point of time, as it is in pathological importance; for, were it otherwise, we are still driven back to the question, what causes the retroversion? If we consult authorities for a reply we are told, in a general way, that it is produced by anything that weakens the uterine supports, or increases the weight of the organ. Now, except in the case of pregnancy—which causes both these effects, and which we are excluding from consideration, as well as the cases in which the accident is produced suddenly—increase in the weight of the womb, and weakening of the uterine supports, imply disease of the organ and its appendages; and those conditions of disease which are found so generally existing with retroversion—so constantly indeed where there are distressing symptoms—are, I believe, not only the causes of those symptoms, but the proximate cause also of the displacement. And this conclusion is strengthened by the fact that

* Jones, Chicago Medical Journal, Jan., 1870.

in nearly every case of chronic retroversion, we shall find, on inquiry, an antecedent history of uterine disease.

What, then, should be the treatment? Clearly to reduce by appropriate means, local and general, the inflammation and engorgement which, while present, must make all attempts to permanently rectify the malposition futile. The remedies which I have found most efficient for this purpose are, local depletion by scarification, the vaginal douche, sedative and astringent injections, and pledgets of cotton wool saturated with glycerine, combined or not with some anodyne or astringent. At the same time we should bear in mind what too many practitioners engaged in any special practice seem much too prone to forget, namely, that all local appliances, whether mechanical, physical or chemical, are to be regarded as auxiliary and subordinate to the general therapeutical treatment required, whether the disease consists in lesion of structure, deviation of place, or disorder of function. There is a tendency, in gynæcology especially, to magnify unduly the importance of local disorders and local remedies, and to overlook, or at least to underrate, the general abnormal condition, and the constitutional means of restoration; and this tendency has invited and received deserved reproach upon this important branch of practice. The great advantage of devotion to any special department of practice, is the acquisition of an accuracy of diagnostic tact which is rarely attained by the general practitioner. But if while cultivating this we are unmindful of the inter-dependence and sympathy of each organ of the body with all the others, we shall inevitably become illiberal and contracted in our views and mere routinists in practice.

Shall we treat directly the malposition? Unquestionably we must if we would effect a cure. Although cases occasionally occur in which the removal of the engorgement and inflammation suffice to restore the organ to its normal position, I believe that they are generally cases in which the disease has existed a comparatively short time, and that in the large majority we shall find ourselves obliged to use direct means to rectify the displacement. We cannot but regard the retroversion as an evil, although not the greatest, perhaps, and we have seen how it may complicate and keep in existence other disorders; and further, why it is that in long-standing cases the displaced organ cannot restore itself.

And, hence, I am disposed to regard any plan of treatment which contemplates only the removal of the accompanying engorgement and inflammation in a case of extreme retroversion, without regard to the rectifying of the positional disorder, as unreliable and incomplete. To my mind such a course seems as unreasonable as it would be to treat a strangulated hernia by the use of leeches and cold applications without attempting any reposition of the bowel.

The mechanical devices by which the retroverted womb is restored and maintained in its place are familiar to you all, and I need not therefore consume your time by detailing them. But I would suggest that the best time for replacing the organ, after the tenderness and heaviness have been sufficiently reduced, is just prior to a menstrual period. If the operation be carefully performed, and great caution exercised during the time of the flow, it will occasionally be followed by permanent relief. The catamenial period determines a reparation and renewal of uterine tissue which supply a valuable aid in the treatment of most of the disorders of the organ.

To summarize, then, I have endeavored to make the following points, viz.:

1. Chronic retroversion of the womb, unaccompanied by structural disease, is of rare occurrence, and may be regarded as a harmless disorder.

2. The distressing symptoms usually attributed to retroversion are dependent upon complications involving structural change, as engorgement, inflammation, retroflexion, etc., rather than upon the displacement itself.

3. These complications are to be regarded as primary in point of time, as they are in pathological importance.

4. The treatment should have reference, first, to the removal of the structural lesion; and secondly, to the replacement of the organ and its retention by appropriate means.

785 Michigan Ave.

